# Editors’ Pick: What a pain…, or not!

**DOI:** 10.1186/2041-2223-5-8

**Published:** 2014-07-01

**Authors:** Manfred Kayser

**Affiliations:** 1 Department of Forensic Molecular Biology, Erasmus MC University Medical Centre Rotterdam, PO Box 2040, Rotterdam, CA 3000, The Netherlands

## 

How nice, I thought as a child, would it be to not feel physical pain. Later I realized that sense of pain may exist for a reason; it was suggested that physical pain has evolved as an alarm system to protect the body from serious damage. Much later I read about human disorders involving severe insensitivity to physical pain. Still, the idea of not feeling pain remains appealing (especially anticipating my coming years when some painful age-related health problems are likely to kick in).

Previously, it was demonstrated that a loss-of-function mutation in the sodium ion channel gene *SCN9A* leads to the inability to experience pain [1]. In a more recent study, Leipold *et al.*[2] identified another sodium ion channel gene, *SCN11A*, which, when harboring a particular missense mutation, causes loss of pain perception. *SCN11A* encodes the voltage-gated sodium ion channel NA_v_1.9, which is highly expressed in nociceptors (i.e., neurons that transmit sensory information from the body periphery to the spinal cord). The authors started out with whole-exome sequencing of healthy German parents and their child diagnosed with the congenital inability to experience pain. A heterozygote non-synonymous mutation in *SCN11A* was identified as the only *de novo* event detected in this affected child. Aiming to confirm this finding in independent samples, the authors performed Sanger sequencing of *SCN11A* exons in 58 additional individuals diagnosed with early-onset severe sensory loss, most of them representing sporadic cases. One Swedish individual showed the very same heterozygous *SCN11A* mutation, and the clinical history of both individuals was also similar. From these findings, the authors hypothesized that the identified mutation that leads to a replacement of leucine with proline (p.Leu811Pro) at the distal end of one of the transmembrane segments in domain II of NA_v_1.9 is disease causing and leads to loss of pain perception.

Seeking molecular proof, the authors introduced the orthologous alteration (Leu799Pro) into the mouse *Scn11a* gene, but found no obvious morphological changes in sensory axons or in nerve fibers in the skin of the viable heterozygote knock-in mice. However, 11% of the knock-in mice had severe lesions that were not observed in the wild-type mice. Since the lesions also appeared in knock-in mice housed solitarily, it was assumed that they were self-inflicted. Also other experimental evidence pointed towards reduced pain sensitivity in these knock-in mice.

Because NA_v_1.9 had been suggested to influence the excitability of dorsal root ganglia (DRG) neurons (i.e., their property to react to stimulation), the authors then investigated the electrical potential of DRG neurons isolated from wild-type and mutant mice. They found that a significant fraction of the mutant channels were active under resting conditions, and that the duration of action potentials was significantly reduced in the mutant neurons compared with wild-type neurons. In knock-out mice, however, only a minor effect on action potentials was observed. Additional experiments showed that the excessive activity at resting conditions caused sustained depolarization of nociceptors, impaired generation of action potentials and aberrant synaptic transmission.

To study the effect of the mutation further, the authors heterologously expressed human NA_v_1.9 and NA_v_1.9 Leu811Pro mutant channels in ND7/23 cells (i.e., a mouse neuroblastoma x rat neuron hybrid cell line). They found that the phenotype of human NA_v_1.9 Leu811Pro mutant channels resembled that of mouse NA_v_1.9 Leu799Pro mutant channels, except in terms of steady-state inactivation. From the weak influence of the mutation on inactivation in the human channel, they concluded a stronger gain-of-function effect in humans than in knock-in mice.

Besides impressively demonstrating the power of whole-exome sequencing to find rare candidate disease causing mutations, this study provides a new gain-of-function view on the molecular basis of pain loss, which was previously linked to loss-of-function mutations such as in the NA_v_1.7 channel encoded by *SCN9A*[1]. Furthermore, this finding may open up new avenues for the development of new painkilling drugs [3].

## Competing interests

The author declares that they have no competing interests.

## References

[B1] Cox JJ Reimann F Nicholas AK Thornton G Roberts E Springell K Karbani G Jafri H Mannan J Raashid Y Al-Gazali L Hamamy H Valente EM Gorman S Williams R McHale DP Wood JN Gribble FM Woods CG An SCN9A channelopathy causes congenital inability to experience pain Nature 2006 444 894 898 10.1038/nature05413 17167479 PMC7212082

[B2] Leipold E Liebmann L Korenke GC Heinrich T Giesselmann S Baets J Ebbinghaus M Goral RO Stödberg T Hennings JC Bergmann M Altmüller J Thiele H Wetzel A Nürnberg P Timmerman V De Jonghe P Blum R Schaible HG Weis J Heinemann SH Hübner CA Kurth I A de novo gain-of-function mutation in SCN11A causes loss of pain perception Nature Genetics 2013 45 11 1399 1404 10.1038/ng.2767 24036948

[B3] Cox JJ Wood JN No pain, more gain Nature Genetics 2013 45 11 1271 1272 10.1038/ng.2810 24165728

